# Sex-specific associations of γ-glutamyltransferase to HDL-cholesterol ratio and the incident risk of cardiovascular disease: three Korean longitudinal cohorts from different regions

**DOI:** 10.3389/fendo.2023.1231502

**Published:** 2023-08-15

**Authors:** Dong Hyuk Jung, Byoungjin Park, Ha Eun Ryu, Yong Jae Lee

**Affiliations:** ^1^ Department of Family Medicine, Yongin Severance Hospital, Yongin-si, Republic of Korea; ^2^ Department of Family Medicine, Yonsei University College of Medicine, Seoul, Republic of Korea; ^3^ Department of Family Medicine, Gangnam Severance Hospital, Seoul, Republic of Korea

**Keywords:** GGT/HDL-C ratio, cardiovascular disease, sex difference, regional discrepancies, cohort study

## Abstract

**Background:**

The combination of gamma-glutamyl transferase (GGT) and high-density lipoprotein cholesterol (HDL-C) (GGT/HDL-C) is a novel noninsulin-based marker for assessing the risk of nonalcoholic fatty liver disease and type 2 diabetes mellitus. However, whether the GGT/HDL-C ratio is related to the risk of incident cardiovascular disease (CVD) risk is not well known. Therefore, we aimed to investigate the longitudinal effect of GGT/HDL-C ratio on incident CVD risk in three large cohorts of Korean men and women.

**Methods:**

Data were assessed from 27,643 participants without CVD from the Korean Genome and Epidemiology Study (KoGES), Health Risk Assessment Study (HERAS), and Korea Health Insurance Review and Assessment (HIRA) (HERAS-HIRA) datasets. The participants were divided into four groups according to the GGT/HDL-C quartiles. We prospectively assessed hazard ratios (HRs) with 95% confidence intervals (CIs) for CVD using multivariate Cox proportional-hazard regression models over a 50-month period following the baseline survey.

**Results:**

During the follow-up period, 949 patients (3.4%; 529 men and 420 women) developed CVD. The HRs of CVD for GGT/HDL-C quartiles 2-4 were 1.36 (95% CI, 0.91–2.02), 1.54 (95% CI, 1.05–2.26), and 1.66 (95% CI, 1.12–2.47) after adjusting for metabolic parameters in women, but GGT/HDL-C did not show a trend toward increases in incident CVD in men. Regional discrepancies were evident in the results; the increase in HR in the metropolitan hospital cohort was more pronounced than that in the urban cohort, and the risk was not increased in the rural cohort.

**Conclusion:**

GGT/HDL-C ratio may be a useful predictive marker for CVD in women. Furthermore, the prevalence of CVD was strongly correlated with the GGT/HDL-C ratio in metropolitan areas, and this correlation was more significant than that observed with GGT or HDL-C in isolation.

## Introduction

Cardiovascular diseases (CVD) refer to a collection of medical conditions that affect blood vessels and the heart. Coronary artery disease (CAD) is a prevalent illness worldwide and has been identified as a significant contributor to deaths, representing up to 30% of all deaths worldwide (1, 2). The development of CVD is related to an unhealthy lifestyle, together with multiple comorbidities, such as obesity, hypertension, dyslipidemia, and diabetes (3). Serum gamma-glutamyl transferase (GGT) has traditionally been used as a marker of liver dysfunction, but it has also been found to participate in the atherosclerotic process and predict cardiovascular risk (4–6). High-density lipoprotein cholesterol (HDL-C), also called the “good cholesterol,” has beneficial properties including anti-inflammatory, anti-thrombotic, and antioxidant properties (7, 8). It is often decreased in individuals with CAD and metabolic syndrome, and is related to insulin resistance(IR) and atherogenic dyslipidemia (9–11). However, HDL-C and CVD have recently shown a U-shaped association in men with hypertension, suggesting some limitations in predicting CVD risk using HDL-C alone in both sexes (12).

Several recent studies have shown that combining GGT with HDL-C can improve the identification of nonalcoholic fatty liver disease (NAFLD) (13–15) and type 2 diabetes mellitus (T2DM) (16–18). The GGT/HDL-C ratio was found to be closely linked to NAFLD and T2DM and was a better predictor than a single indicator. NAFLD and T2DM share similar pathophysiological mechanisms, including inflammation and IR (19, 20). These mechanisms are also associated with an increased risk of CVD (21). However, no longitudinal cohort study has yet investigated the relationship between the GGT/HDL-C ratio and CVD risk. The majority of CVD can be avoided by addressing modifiable risk factors such as smoking, excessive alcohol consumption, obesity, unhealthy diet, and physical inactivity (22, 23). Early detection of CVD is crucial for initiating treatment with lifestyle interventions and medications. In this study, we investigated the relationship between GGT/HDL-C ratio and the incidence of CVD over time in Korean individuals.

## Materials and methods

### Study design and participants

**Figure 1:**
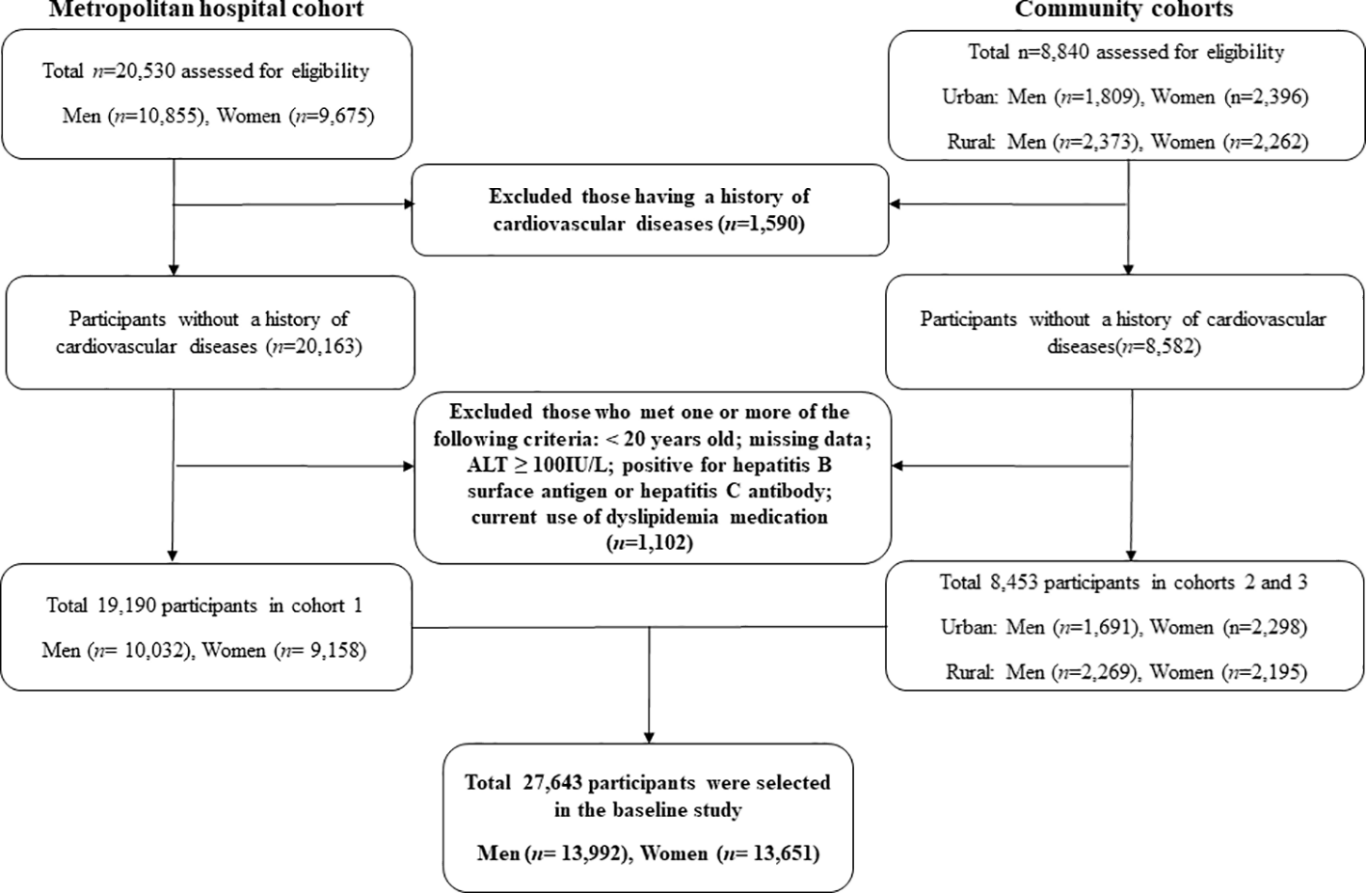
Flowchart for the selection of study participants.

Two types of data were used. The first set of data was obtained from the Korean Genome and Epidemiology Study (KoGES), which has been extensively described in terms of design and methodology. This longitudinal study aimed to evaluate the prevalence, incidence, and risk factors associated with chronic degenerative disorders, including diabetes, hypertension, osteoporosis, and cardiovascular diseases. The KoGES cohort comprised 10,030 participants residing in both urban (Ansan) and rural (Ansung) areas. The participants were recruited during a baseline survey conducted between 2001 and 2002. Subsequently, biennial surveys were conducted until the sixth follow-up (2013-2014. Second, we used data from the Health Risk Assessment Study (HERAS) and Korea Health Insurance Review and Assessment (HIRA), which were also described previously. In brief, the cohort consisted of 20,530 subjects who visited the Health Promotion Center at the Yonsei University Gangnam Severance Hospital for health examinations. The majority of the participants resided in the metropolitan Gangnam area of Seoul. The baseline survey for this cohort was conducted between November 2006 and June 2010, and participants were assessed over a period of 50 months from the time of enrollment. Subjects meeting any of the following criteria were excluded: previous diagnosis of CVD, under 20 years of age, missing data, currently using lipid-lowering medication, and ALT ≥100 mg/L: hepatitis B surface antigen or hepatitis C antibody. Finally, 27,643 participants without CVD were analyzed in this study (**Figure 1**). Informed consent was obtained from all the eligible participants. This study was approved by the Institutional Review Board (IRB) of Yongin Severance Hospital (IRB number:9-2020-0018).

### Data collection

Each participant completed a comprehensive questionnaire that captured information regarding their lifestyle and medical history. Smoking status was categorized as never smoked, ex-smoker, or current smoker. Regular alcohol consumption was defined as consuming more than 140 g of alcohol per week based on the frequency of alcohol consumption reported by the subjects. Body weight and height measurements were taken while participants wore light indoor clothing and no shoes, with a precision of 0.1 kg and 0.1 cm, respectively. Body mass index (BMI) was calculated as weight divided by height in meters squared (kg/m2). Systolic blood pressure (SBP) and diastolic blood pressure (DBP) were measured in the sitting position after 10 min of rest using a standard mercury sphygmomanometer (Baumanometer; W.A. Baum Co Inc., Copiague, NY, USA). The mean arterial pressure was derived from SBP and DBP values. Hypertension was defined as SBP ≥ 140 mmHg, DBP ≥ 90 mmHg, or the current use of hypertension medication. After a 12-h overnight fast, blood samples were collected from the subjects through the antecubital vein. Concentrations of total cholesterol, triglyceride, high-density lipoprotein cholesterol (HDL-C), plasma glucose level, aspartate aminotransferase (AST), alanine aminotransferase (ALT), and GGT were measured enzymatically using a Chemistry Analyzer (Hitachi 7600, Tokyo, Japan by August 2002 and ADVIA 1650, Siemens, Tarrytown, NY from September 2002). C-reactive protein (CRP) concentration was measured using an immunoradiometric assay (ADVIA 1650, Siemens, Tarrytown, NY, USA).

### Study outcomes

The primary outcome of our study, as previously described, was ischemic heart disease (IHD), which encompasses angina pectoris (ICD-10 code I20) or acute myocardial infarction (ICD-10 code I21) that occurred after participants’ enrollment into the study. To establish baseline and post-survey outcomes, we utilized a personal 13-digit identification number assigned to each participant by the Korean Health Insurance Review and Assessment Service (HIRA) between November 1, 2006, and December 31, 2010. For the KoGES cohort, the self-reported CVD status of the participants was obtained through a questionnaire.

### Statistical analysis

We categorized the participants into four groups based on their baseline GGT-HDL levels. Since men had higher levels of GGT/HDL-C than women, we performed all analyses separately for men and women. The cutoff levels of GGT/HDL-C were 0.43, 0.67 and 1.13 in men and 0.21, 0.28 and 0.41 in women. All data are presented as the mean ± standard deviation or percentage. According to the GGT/HDL-C quartiles, the baseline characteristics of the study population were compared using Pearson’s chi-square test for categorical variables and an analysis of variance (ANOVA) model for continuous variables. In the multivariate analysis, hazard ratios (HRs) and 95% confidence intervals (CIs) for incident CVD were calculated using the Cox proportional hazards regression model after adjusting for potential confounding variables. All analyses were performed using the SAS version 9.2 software (SAS Institute Inc., Cary, NC, USA). All statistical tests were two-sided, and P values <0.05 were considered statistically significant.

## Results

### Baseline characteristics

The current study included 27,643 subjects from three Korean longitudinal cohorts from different regions, with a CVD incidence of 949 individuals (3.4%; 529 men and 420 women) up to a 10-year follow-up. **Table 1** shows the biochemical and demographic characteristics of 13,992 men and 13,651 women according to the GGT/HDL-C ratio quartile. The mean age and GGT/HDL-C ratios were 47.4 ± 10.5 years and 0.98 ± 1.22 for men, and 47.6 ± 10.9 years and 0.38 ± 0.48 in women, respectively. The group with the fourth GGT/HDL-C quartile showed the highest mean values of BMI, arterial pressure, fasting plasma glucose, total cholesterol, liver function tests, and C-reactive protein, both in men and women. The proportion of alcohol drinking in men significantly increased according to the GGT/HDL-C quartiles; in contrast, those in women did not exhibit significant trends.

**Table 1:** Baseline characteristics of the study population according to the GGT/HDL-C quartiles in men and women.

Men	Overall (n = 13,992)		Q1 (n = 3,467)	Q2 (n = 3,554)	Q3 (n = 3,494)	Q4 (n = 3,477)	*P-value* ^a^	*Post hoc* ^b^
GGT/HDL-C	0.98 ± 1.22		≤ 0.43	0.44 – 0.67	0.68 – 1.12	≥ 1.13		
Age (years)	47.4 ± 10.5		47.0 ± 12.0	47.6 ± 10.5	47.8 ± 9.9	47.4 ± 9.4	0.004	a,b
Body mass index (kg/m^2^)	22.7 ± 4.0		21.8 ± 3.4	22.7 ± 3.8	23.1 ± 4.2	23.2 ± 4.6	< 0.001	a,b,c,d,e
Current smoker (%)	40.4		38.7	40.9	41.1	40.8	< 0.001	–
Alcohol drinking (%)	47.5		38.5	41.6	48.9	61.0	< 0.001	–
Systolic blood pressure (mmHg)	125.3 ± 15.6		121.5 ± 15.2	124.4 ± 14.9	126.4 ± 15.6	128.9 ± 15.8	< 0.001	a,b,c,d,e,f
Diastolic blood pressure (mmHg)	79.9 ± 10.2		76.8 ± 9.7	79.2 ± 9.8	80.9 ± 10.0	82.9 ± 10.2	< 0.001	a,b,c,d,e,f
Fasting plasma glucose (mg/dl)	96.6 ± 22.5		91.8 ± 16.5	95.0 ± 19.3	97.3 ± 21.7	102.4 ± 29.3	< 0.001	a,b,c,d,e,f
Total cholesterol (mg/dl)	193.0 ± 34.6		184.7 ± 31.0	190.9 ± 32.8	195.8 ± 33.6	200.8 ± 38.5	< 0.001	a,b,c,d,e,f
Triglyceride (mg/dl)	153.9 ± 110.2		97.2 ± 44.9	129.3 ± 62.6	167.3 ± 93.0	222.0 ± 159.9	< 0.001	a,b,c,d,e,f
HDL-cholesterol (mg/dl)	48.7 ± 11.3		54.8 ± 11.5	48.4 ± 10.3	45.8 ± 10.1	45.7 ± 10.6	< 0.001	a,b,c,d,e
Aspartate aminotransferase (IU/L)	24.3 ± 10.6		20.5 ± 6.3	21.9 ± 6.7	24.1 ± 7.9	30.9 ± 15.4	< 0.001	a,b,c,d,e,f
Alanine aminotransferase (IU/L)	26.9 ± 15.0		18.1 ± 7.5	22.7 ± 9.8	28.4 ± 13.3	38.7 ± 18.4	< 0.001	a,b,c,d,e,f
γ-glutamyltransferase (IU/L)	45.6 ± 54.2		17.4 ± 4.2	26.3 ± 6.4	39.3 ± 10.3	99.7 ± 86.7	< 0.001	a,b,c,d,e,f
C-reactive protein (mg/L)	1.3 ± 3.6		0.8 ± 2.1	1.1 ± 2.9	1.4 ± 3.5	1.8 ± 5.1	< 0.001	a,b,c,d,e,f
Hypertension (%)	12.4		7.8	11.4	13.6	16.7	< 0.001	–
Diabetes mellitus (%)	12.0		7.4	10.3	13.2	17.2	< 0.001	–
Women	Overall (n = 13,651)	Q1 (n = 3,574)		Q2 (n = 3,370)	Q3 (n = 3,313)	Q4 (n = 3,394)	*P-value* ^a^	*Post hoc* ^b^
GGT/HDL-C	0.38 ± 0.48	≤ 0.21		0.22 – 0.28	0.29 – 0.41	≥ 0.42		
Age (years)	47.6 ± 10.9	43.0 ± 10.1		46.4 ± 10.8	49.6 ± 10.4	51.8 ± 10.0	< 0.001	a,b,c,d,e,f
Body mass index (kg/m^2^)	22.3 ± 3.7	21.1 ± 2.8		21.8 ± 3.5	22.8 ± 3.8	23.6 ± 4.0	< 0.001	a,b,c,d,e,f
Current smoker (%)	3.8	3.4		3.1	3.9	4.9	< 0.001	–
Alcohol drinking (%)	21.3	20.0		21.4	21.5	22.2	0.180	–
Systolic blood pressure (mmHg)	118.3 ± 17.4	113.4 ± 14.8		116.4 ± 16.6	119.8 ± 18.0	123.9 ± 18.1	< 0.001	a,b,c,d,e,f
Diastolic blood pressure (mmHg)	74.7 ± 11.1	71.0 ± 9.6		73.6 ± 10.6	75.8 ± 11.3	78.6 ± 11.3	< 0.001	a,b,c,d,e,f
Fasting plasma glucose (mg/dl)	90.5 ± 16.9	86.6 ± 9.2		88.4 ± 13.8	91.0 ± 16.7	96.4 ± 23.5	< 0.001	a,b,c,d,e,f
Total cholesterol (mg/dl)	191.1 ± 35.5	184.8 ± 30.7		186.7 ± 34.2	192.1 ± 35.6	201.0 ± 38.8	< 0.001	b,c,d,e,f
Triglyceride (mg/dl)	111.9 ± 72.8	75.1 ± 28.6		94.7 ± 42.5	118.9 ± 60.5	160.9 ± 104.4	< 0.001	a,b,c,d,e,f
HDL-cholesterol (mg/dl)	55.6 ± 12.9	65.8 ± 11.1		56.4 ± 10.2	51.2 ± 10.9	48.2 ± 11.8	< 0.001	a,b,c,d,e,f
Aspartate aminotransferase (IU/L)	20.4 ± 7.8	17.9 ± 4.7		19.0 ± 6.1	20.2 ± 6.1	24.6 ± 11.1	< 0.001	a,b,c,d,e,f
Alanine aminotransferase (IU/L)	17.6 ± 10.3	13.1 ± 5.2		14.7 ± 6.5	17.3 ± 8.0	25.5 ± 14.1	< 0.001	a,b,c,d,e,f
γ-glutamyltransferase (IU/L)	19.9 ± 23.3	11.1 ± 2.0		13.7 ± 2.5	17.1 ± 3.9	37.8 ± 41.5	< 0.001	a,b,c,d,e,f
C-reactive protein (mg/L)	0.8 ± 2.8	0.6 ± 1.9		0.6 ± 1.6	0.8 ± 2.2	1.3 ± 4.6	< 0.001	b,c,d,e,f
Hypertension (%)	9.1	3.1		5.7	9.8	18.2	< 0.001	–
Diabetes mellitus (%)	11.0	3.0		8.0	14.0	19.4	< 0.001	–

^a^P-values were calculated using 1-way ANOVA or Pearson’s chi-square test. ^b^Post hoc analysis with the Bonferroni method: a, Q1 versus Q2; b, Q1 versus Q3; c, Q1 versus Q4; d, Q2 versus Q3; e, Q2 versus Q4; and f, Q3 versus Q4.

### Sex-specific hazard ratios for CVD

**Table 2** shows the results of the multivariate Cox proportional hazards regression analysis for predicting new-onset CVD according to GGT/HDL-C quartiles. In men, the HRs of incident CVD diabetes were 1.34 (95% CI, 1.05–1.73) in the fourth quartile of GGT/HDL-C after adjusting for age and BMI. We also assessed the longitudinal associations between the GGT/HDL-C ratio and the risk of CVD incidence after additional adjustment for lifestyle factors, including alcohol intake and major metabolic parameters, such as mean arterial blood pressure, fasting plasma glucose, total cholesterol, alanine aminotransferase, C-reactive protein, hypertension, and diabetes mellitus (models 2 and 3). The GGT/HDL-C ratio did not show an increasing trend for the incidence of CVD. Meanwhile, in women, CVD risk in the group with the highest quartile of GGT/HDL-C significantly increased by 83%, 69%, and 66% in models 1, 2, and 3, respectively, compared with the reference quartile (P = 0.001, P = 0.029, P = 0.011, respectively).

**Table 2:** Hazard ratios and 95% confidence intervals for cardiovascular diseases according to GGT/HDL-C quartiles in men and women.

GGT/HDL-C quartiles							
Men			Q1 (≤ 0.43)	Q2 (0.44 – 0.67)	Q3 (0.68 – 1.12)	Q4 (≥ 1.13)	*P* for trend
New cases of ischemic heart disease,n			113	119	147	150	
Mean follow-up, years			3.7 ± 3.0	3.7 ± 3.0	3.8 ± 3.1	3.9 ± 3.3	
Pearson-years of follow-up			12,800	13,131	13,413	13,564	
Incidence rate/1000 person –years			8.8	9.1	10.4	11.1	
Model 1	HR (95% CI)		1.00 (reference)	1.10 (0.85–1.44)	1.34 (1.04–1.72)	1.34 (1.05–1.73)	0.050
	*P-*value		–	0.469	0.023	0.021	
Model 2	HR		1.00 (reference)	1.06 (0.81–1.39)	1.32 (1.01–1.71)	1.26 (0.93–1.69)	0.164
	*P*-value		–	0.663	0.041	0.135	
Model 3	HR		1.00 (reference)	1.04 (0.79–1.35)	1.25 (0.96–1.63)	1.21 (0.90–1.63)	0.315
	*P*-value		–	0.793	0.100	0.216	
Women			Q1 (≤ 0.21)	Q2 (0.22 – 0.28)	Q3 (0.29 – 0.41)	Q4 (≥ 0.42)	*P* for trend
New cases of ischemic heart disease,n			43	84	132	161	
Mean follow-up, years			3.2 ± 2.7	4.1 ± 3.4	4.3 ± 3.6	4.3 ± 3.5	
Pearson-years of follow-up			11,518	13,652	14,201	14,581	
Incidence rate/1000 person –years			3.7	6.2	9.3	11.0	
Model 1	HR (95% CI)		1.00 (reference)	1.34 (0.90–1.98)	1.66 (1.14–2.42)	1.83 (1.26–2.64)	0.005
	*P-*value		–	0.151	0.008	0.001	
Model 2	HR		1.00 (reference)	1.35 (0.91–2.01)	1.53 (1.04–2.25)	1.69 (1.14–2.25)	0.059
	*P*-value		–	0.141	0.029	0.029	
Model 3	HR		1.00 (reference)	1.36 (0.91–2.02)	1.54 (1.05–2.26)	1.66 (1.12–2.47)	0.073
	*P*-value		–	0.133	0.027	0.011	

Model 1: adjusted for age and body mass index.

Model 2: adjusted for age, body mass index, smoking status, alcohol intake, mean arterial blood pressure, fasting plasma glucose, total cholesterol, alanine aminotransferase, and C-reactive protein.

Model 3: adjusted for age, body mass index, smoking status, alcohol intake, mean arterial blood pressure, fasting plasma glucose, total cholesterol, alanine aminotransferase, C-reactive protein, hypertension, and diabetes mellitus.

### Subgroup analysis for incident CVD

**Table 3** shows the multivariate Cox proportional hazards regression models for incident CVD according to GGT and HDL-C levels and their ratios. After setting the lowest GGT quartile as a reference group, the highest GGT quartile showed no significant increase in CVD risk in men, while there was a borderline significant increase in women (HR (95% CI) = 1.13 [0.84–1.52] and 1.40 [0.99-1.97], respectively). On the other hand, in multivariate analysis, the risk for incident CVD was decreased only in men but not in women according to the HDL-C quartile (HR (95% CI) = 0.70 [0.54–0.91] and 0.95 [0.69-1.31], respectively). Lastly, compared with the reference GGT/HDL-C ratio quartile, the HR of incident CVD for the fourth quartile increased after adjusting for potential confounding variables only in women, but not in men (HR (95% CI) = 1.66 [1.12–2.47] and 1.20 [0.90-1.60], respectively). Furthermore, we evaluated whether the GGT/HDL-C ratio affected the CVD risk in different regional cohorts (**Table 4**). The highest GGT/HDL ratio quartile in men did not increase CVD risk in all cohorts. In women, the increase in HR in the metropolitan hospital cohort was more pronounced than that in the urban cohort, and the risk was not increased in the rural cohort. This finding may indicate regional differences in our cohort based on several factors.

**Table 3:** Multivariate Cox proportional-hazards regression models for incident cardiovascular disease according to GGT, HDL-C, and their ratio.

	GGT		HDL-C		GGT/HDL-C	
	Men	Women	Men	Women	Men	Women
Age (years)	1.05 (1.04–1.06)	1.07 (1.05–1.08)	1.05 (1.04–1.06)	1.07 (1.05–1.08)	1.05 (1.04–1.06)	1.06 (1.05–1.08)
Body mass index (kg/m^2^)	1.10 (1.07–1.13)	0.99 (0.96–1.03)	1.10 (1.07–1.13)	0.99 (0.96–1.03)	1.10 (1.07–1.13)	0.99 (0.96–1.03)
Current smoker (%)	1.43 (1.11–1.83)	1.54 (0.96–2.45)	1.43 (1.12–1.84)	1.56 (0.98–2.49)	1.41 (1.10–1.81)	1.53 (0.96–2.45)
Alcohol drinking (%)	0.70 (0.57–0.85)	0.74 (0.56–0.98)	0.75 (0.62–0.90)	0.76 (0.57–1.01)	0.70 (0.58–0.85)	0.75 (0.56–0.99)
Mean arterial pressure (mmHg)	1.00 (0.99–1.01)	1.01 (1.00–1.02)	1.00 (0.99–1.01)	1.01 (1.00–1.02)	1.00 (0.99–1.01)	1.01 (1.00–1.02)
Fasting plasma glucose (mg/dl)	1.00 (1.00–1.01)	1.00 (1.00–1.01)	1.00 (1.00–1.01)	1.00 (1.00–1.01)	1.00 (1.00–1.01)	1.00 (1.00–1.01)
Total cholesterol (mg/dl)	1.00 (1.00–1.01)	1.00 (1.00–1.01)	1.00 (1.00–1.01)	1.00 (1.00–1.01)	1.00 (1.00–1.01)	1.00 (1.00–1.01)
Alanine aminotransferase (IU/L)	1.00 (0.99–1.00)	0.99 (0.98–1.01)	1.00 (0.99–1.00)	1.00 (0.99–1.01)	1.00 (0.99–1.00)	0.99 (0.98–1.01)
C-reactive protein (mg/L)	1.01 (0.99–1.03)	1.00 (0.97–1.04)	1.01 (0.99–1.03)	1.00 (0.97–1.04)	1.01 (0.99–1.03)	1.00 (0.97–1.04)
Hypertension (%)	1.74 (1.38–2.19)	1.70 (1.24–2.31)	1.73 (1.37–2.17)	1.73 (1.27–2.36)	1.74 (1.38–2.18)	1.71 (1.25–2.33)
Diabetes (%)	1.29 (1.01–1.65)	0.94 (0.71–1.25)	1.28 (1.01–1.64)	0.95 (0.71–1.26)	1.29 (1.01–1.65)	0.93 (0.70–1.24)
Q4 vs Q1	1.13 (0.84–1.52)	1.40 (0.99–1.97)	0.70 (0.54–0.91)	0.95 (0.69–1.31)	1.20 (0.90–1.60)	1.66 (1.12–2.47)

**Table 4:** Multivariate Cox proportional-hazards regression models for incident cardiovascular disease according to GGT/HDL-C quartiles in different cohorts.

	Metropolitan hospital		Urban community		Rural community	
	Men	Women	Men	Women	Men	Women
Age (years)	1.06 (1.05–1.08)	1.06 (1.04–1.08)	1.09 (1.03–1.15)	1.05 (1.00–1.10)	1.04 (0.98–1.11)	1.14 (1.05–1.24)
Body mass index (kg/m^2^)	1.07 (1.01–1.12)	1.01 (0.95–1.08)	0.96 (0.85–1.08)	0.99 (0.91–1.07)	1.11 (0.96–1.28)	0.89 (0.75–1.05)
Current smoker (%)	1.45 (1.03–2.04)	0.49 (0.12–1.99)	1.21 (0.72–2.02)	1.63 (0.89–2.98)	1.57 (0.92–2.68)	6.20 (2.40–16.10)
Alcohol drinking (%)	0.87 (0.66–1.14)	0.80 (0.45–1.44)	0.71 (0.47–1.08)	0.81 (0.55–1.20)	0.84 (0.55–1.30)	0.80 (0.44–1.47)
Mean arterial pressure (mmHg)	0.99 (0.98–1.01)	0.99 (0.97–1.01)	0.99 (0.97–1.01)	1.01 (1.00–1.03)	1.01 (0.99–1.03)	1.00 (0.98–1.02)
Fasting plasma glucose (mg/dl)	1.01 (1.00–1.01)	1.00 (0.99–1.01)	1.00 (1.00–1.01)	1.00 (0.99–1.01)	1.00 (0.99–1.01)	1.00 (0.99–1.01)
Total cholesterol (mg/dl)	1.00 (1.00–1.01)	1.01 (1.00–1.01)	1.00 (1.00–1.01)	1.00 (1.00–1.01)	1.01 (1.00–1.01)	1.00 (1.00–1.01)
Alanine aminotransferase (IU/L)	1.00 (0.99–1.01)	0.99 (0.97–1.01)	0.99 (0.97–1.01)	1.00 (0.98–1.01)	0.99 (0.98–1.01)	0.99 (0.97–1.02)
C-reactive protein (mg/L)	1.00 (0.97–1.03)	0.96 (0.88–1.05)	0.99 (0.78–1.27)	1.00 (0.82–1.20)	1.22 (0.83–1.80)	0.93 (0.42–2.08)
Hypertension (%)	1.56 (1.16–2.09)	2.25 (1.43–3.56)	1.65 (0.93–2.91)	1.05 (0.60–1.85)	1.89 (1.05–3.40)	2.10 (0.93–4.75)
Diabetes (%)	0.97 (0.59–1.61)	0.96 (0.40–2.28)	2.17 (1.30–3.61)	0.94 (0.61–1.43)	1.39 (0.81–2.37)	1.93 (0.98–3.80)
Q4 vs Q1	1.07 (0.70–1.63)	2.59 (1.34–5.02)	1.69 (0.95–3.03)	1.79 (0.96–3.33)	0.88 (0.44–1.75)	0.81 (0.33–1.97)

## Discussion

In this cohort study, we aimed to investigate the association between GGT/HDL-C ratio and CVD risk. The GGT/HDL-C ratio was positively correlated with the prevalence of CVD in women regardless of the main cardiometabolic indices. According to the GGT/HDL-C quartiles, the HRs of overt CVD in women increased considerably; however, those in men did not show any significant increase. In addition, the correlation between the GGT/HDL-C ratio and the prevalence of CVD was strong in metropolitan areas, and it outperformed GGT and HDL-C alone.

CVD is a leading cause of mortality worldwide with significant health and economic consequences (1). Several indicators have been proposed for predicting CVD (24). GGT exists on the extracellular surface and plays a role in the cleavage of glutathione when oxidative stress increases (4). GGT indirectly affects LDL cholesterol oxidation and atherogenesis (25, 26). Numerous studies have investigated the association between GGT and CVD because of its pro-inflammatory nature. These studies have demonstrated a positive association between GGT and metabolic syndrome as well as CVD (5, 27, 28). HDL-C is widely established to have an inverse relationship with CVD by modulating inflammation and controlling cholesterol outflow from tissues (29–31).

An increase in the GGT/HDL-C ratio suggests an increase in GGT levels or a reduction in HDL-C levels. Based on previous studies, it is expected that the GGT/HDL-C ratio correlates with CVD risk prediction. The GGT/HDL-C ratio has emerged as a potential biomarker for NAFLD and T2DM. Several recent studies have investigated the association between GGT/HDL-C ratio and NAFLD. For example, a study conducted by Xie et al. revealed a positive and nonlinear association between GGT/HDL-C ratio and the risk of developing NAFLD in non-obese Chinese individuals (13). Similarly, the relationship between GGT/HDL-C ratio and T2DM has also been investigated in other studies (16–18). Studies have indicated a positive association between GGT/HDL-C ratio and T2DM incidence, suggesting its potential as a biomarker for T2DM risk. To the best of our knowledge, no published studies have investigated the relationship between GGT/HDL-C ratio and the incidence of CVD. For this reason, we carried out the current investigation, and our results revealed a significant positive correlation between the GGT/HDL-C ratio and CVD incidence in women. Furthermore, previous studies have shown that the GGT/HDL-C ratio exhibits a more significant correlation with NAFLD (13–15) or T2DM (17, 18) than GGT or HDL alone. These findings were consistent with the results of the present study. Moreover, a recent epidemiological study also reported that high HDL-C can increase CVD risk and may have sex differences (12). It deserves clinical consideration that there may be limitations in evaluating CVD risk using HDL-C alone.

We also found that the association between the GGT/HDL-C ratio and CVD was significant only in women, and differences between men and women were observed in alcohol consumption. GGT is primarily regarded as a marker of liver function and alcohol consumption (32). In a previous study, the association between GGT and CAD was stronger in drinkers than in nondrinkers (33). In a study conducted by Hozawa et al. in Japan, a cohort of 2724 men and 4122 women was followed up for 9.6 years to assess CVD mortality. The results showed that increased GGT levels were associated with a higher HR for CVD-related death of 2.88 in women, but no significant association was found in men (34). The prevalence of alcohol consumption among East Asian women was lower than that among men. After adjusting for alcohol consumption, there was no association between GGT and CVD mortality in men. These findings suggest that GGT may have specific CVD risks, regardless of alcohol consumption, and the sex-specific results are consistent with our findings. It is expected that the GGT/HDL-C ratio can be a reliable predictor of CVD in women unaffected by alcohol consumption.

Another notable aspect of this study is the observed regional variation. Some explanations can be considered for the observed connections. Oh et al. conducted a study to explore the geographical variations and influential factors of cardiometabolic disease prevalence in 230 administrative districts in South Korea (35). They determined whether the standardized prevalence of cardiometabolic diseases (hypertension, stroke, and T2DM) was spatially clustered. The marriage rate, stress-related variables, and wealth status were identified as influential factors. The higher prevalence of CVD in metropolitan areas observed in our study may be attributed to regional differences based on these influential factors. This suggests that the risk of CVD may be higher in metropolitan areas based on the characteristics of the regions investigated in our study. Further studies including incidence data tailored to the regional characteristics of our country are needed to better understand this relationship.

This study is the first to investigate the association between an emerging predictive marker, the GGT/HDL-C ratio, and the risk of CVD. While previous studies have examined the relationship between the GGT/HDL-C ratio and NAFLD and T2DM, research on CVD has been limited. Therefore, the easily accessible GGT/HDL-C ratio is expected to be a useful tool for predicting and preventing CVD in metropolitan women. Additionally, this study has the potential to offer valuable epidemiological data for public health purposes, such as the use of scoring ratios as preventive measures for CVD (36). However, additional research is necessary to establish credible and timely data regarding disease occurrence, which can effectively aid primary disease prevention or mitigation within the field of public health (37).

The strength of our study is that we utilized a longitudinal cohort study with a large sample size of Korean individuals, linked to HERAS and HIRA data, which are derived from the universal coverage system in Korea. This minimized the likelihood of missing data. However, our study had several limitations that need to be acknowledged. We did not take into account certain comorbidities such as peripheral artery disease, atrial fibrillation, thyroid diseases, and NAFLD. Additionally, a detailed assessment of alcohol consumption was not conducted as these variables were not measured at the beginning of the study. Hence, further research is needed to investigate the longitudinal association between the GGT/HDL-C ratio and CVD while considering additional history and lifestyle factors. Furthermore, it should be noted that the HERAS-HIRA dataset only assessed newly developed IHD and did not include calcium score data or coronary angiography. Further studies are warranted to investigate these aspects.

## Conclusions

The GGT/HDL-C ratio was positively correlated with the prevalence of CVD in women compared to GGT or HDL-C alone. The GGT/HDL-C ratio is predicted to be a reliable indicator of CVD in women unaffected by alcohol consumption. Regional disparities were evident in the results, with more pronounced differences observed in the metropolitan areas.

## Data availability statement

The raw data supporting the conclusions of this article will be made available by the authors, without undue reservation.

## Ethics statement

The studies involving human participants were reviewed and approved by Institutional Review Board of Yonsei University College of Medicine. The patients/participants provided their written informed consent to participate in this study.

## Author contributions

DJ, BP, HR, and YL: study concept and design. DJ and BP: acquisition, analysis, and interpretation of data. DJ, BP, and HR: drafting of the manuscript: YL: critical revision of the manuscript for important intellectual content. All authors: contributed to the article and approved the submitted version.
